# Fault-Tolerant Collaborative Control of Four-Wheel-Drive Electric Vehicle for One or More In-Wheel Motors’ Faults

**DOI:** 10.3390/s25051540

**Published:** 2025-03-01

**Authors:** Han Feng, Yukun Tao, Jianbo Feng, Yule Zhang, Hongtao Xue, Tiansi Wang, Xing Xu, Peng Chen

**Affiliations:** 1 School of Automotive and Traffic Engineering, Jiangsu University, Zhenjiang 212013, China; fengh@stmail.ujs.edu.cn (H.F.); taoyk@stmail.ujs.edu.cn (Y.T.); wangtiansi_hit@163.com (T.W.); 2 International Joint Laboratory on Mobility Equipment and Artificial Intelligence for IT Operations, Zhenjiang 212000, China; xuxing@ujs.edu.cn; 3 The 60th Research Institute of CRTC, Nanjing 210016, China; 4 Anhui Jianghuai Automobile Group Corp., Ltd., Hefei 230022, China; zyl.pk@jac.com.cn; 5 Automotive Engineering Research Institute, Jiangsu University, Zhenjiang 212013, China; 6 Graduate School of Bioresources, Mie University, Tsu 514-8507, Japan

**Keywords:** four-wheel drive electric vehicle, in-wheel motor, fault-tolerant collaborative control, multi-system collaboration distribution

## Abstract

A fault-tolerant collaborative control strategy for four-wheel-drive electric vehicles is proposed to address hidden safety issues caused by one or more in-wheel motor faults; the basic design scheme is that the control system is divided into two layers of motion tracking and torque distribution, and three systems, including driving, braking, and front-wheel steering are controlled collaboratively for four-wheel torque distribution. In the layer of motion tracking, a vehicle model with two-degree-of-freedom is employed to predict the control reference values of the longitudinal force and additional yaw moment required; four types of sensors, such as wheel speed, acceleration, gyroscope, and steering wheel angle, are used to calculate the actual values. At the torque distribution layer, SSOD and MSCD distribution schemes are designed to cope with two operating conditions, namely sufficient and insufficient output capacity after local hub motor failure, respectively, focusing on the objective function, constraints, and control variables of the MSCD control strategy. Finally, two operating environments, a straight-line track, and a DLC track, are set up to verify the effectiveness of the proposed control method. The results indicate that, compared with traditional methods, the average errors of the center of mass sideslip angle and yaw rate are reduced by at least 12.9% and 5.88%, respectively, in the straight-line track environment. In the DLC track environment, the average errors of the center of mass sideslip angle and yaw rate are reduced by at least 6% and 4.5%, respectively. The proposed fault-tolerant controller ensures that the four-wheel-drive electric vehicle meets the requirements of handling stability and safety under one or more hub motor failure conditions.

## 1. Introduction

Given the severe environmental pollution and the growing imbalance between petroleum supply and demand, accelerating the development of new energy vehicles has become an urgent necessity. Many countries have introduced policies to promote the development of energy-saving vehicles and the technological transformation of pure electric drive, the related in-wheel motor technology is one of the disruptive technologies in the automotive industry. Four in-wheel motors are mounted separately into the wheels to drive the vehicle together, which is called a four-wheel-drive electric vehicle (4WDEV). It has the absolute advantages of high efficiency, fast response, and a drive-by-wire system [1,2,3] that has been recognized as the ideal configuration for future electric vehicles. However, the ever-changing driving conditions and complex operating environments encountered by vehicles can subject the in-wheel motors within the wheels to intermittent and strong impacts [4,5], and the long-time service can lead in-wheel motors to malfunction. Once an in-wheel motor is faulty, the vehicle inevitably generates unexpected yaw moments [6,7,8]. When significant yaw moments are required, it is bound to consume more longitudinal forces of the tire [9,10] and reduce the vehicle’s stability margin. In particular, the tire’s lateral force may exceed the self-regulation capability [11,12]. This greatly increases the risk of losing control of the vehicle [13,14,15,16]. Therefore, it is highly significant to design a fault-tolerant controller.

In recent years, numerous researchers have explored fault diagnosis and safety assessment of in-wheel motors, achieving significant advancements [17]. The intersection projection method has been proposed for fault diagnosis of external rotor permanent magnet synchronous motor (ER-PMSM) based on the total harmonic distortion of the no-load back electromotive force of a single motor, which is used to diagnose the eccentricity position of ER-PMSM static eccentricity (SE) faults [18]. The assessment model has been constructed to evaluate the operational safety of in-wheel motors based on picture fuzzy set theory, in particular with multiple symptom parameters in successive stages, and then achieve a comprehensive evaluation of the operational safety of in-wheel motors [19,20]. The load demodulation and normalization method without vibration sensors has been proposed for fault detection and diagnosis of in-wheel motor transmission components under variable load conditions; the dominant idea is that the controller’s electrical signal is used to replace the vibration sensor for diagnosing the bearing fault of in-wheel motor [21,22,23,24]. Distributed fault-tolerant consensus tracking control is proposed for multi-agent systems with abrupt and incipient actuator faults under fixed and switching topologies [25]. Artificial hydrocarbon networks and support vector data description are used to construct an intelligent diagnosis system for mechanical faults of in-wheel motors, which includes sequential diagnosis and one-stop diagnosis, the ultimate objective is to promote the operational safety of 4WDEV [26,27,28,29]. A new method for adaptive separation of multi-source signals and extraction of composite fault features is proposed for composite fault detection in wheel motor bearings. The method integrates blind deconvolution based on blind source separation to address the challenge of extracting weak features [30]. The above research has focused on fault diagnosis of in-wheel motors; these have laid a basis for fault degree identification and operational safety assessment.

However, ensuring the safe operation of in-wheel motors or vehicles remains a critical issue [31,32,33,34]. The multi-objective optimization method based on torque allocation optimization has been proposed to improve the overall efficiency and operational safety of the drive system of distributed drive electric vehicles. The objective function was established by combining the drive system efficiency and the required torque value of the distributed drive electric system and solved by NSGA-II and HGTSA [35]. The multi-model fault-tolerant control system has been introduced to provide excellent fault-tolerant performance under various operating conditions. In this approach is that multiple fault-tolerant controllers were designed for various typical operating modes, and the weighted signals from each fault-tolerant controller were used to calculate the output of the multi-model control system [36]. Cooperative game theory has been used to present a fault-tolerant control method for treating the problem of the four in-wheel motors as a cooperative game, effectively ensuring vehicle stability in various actuator fault scenarios [37]. The active fault-tolerant control method has been proposed that compensates for modeling errors using a nonlinear disturbance observer for ensuring vehicle stability and improving tracking performance of four-wheel steering electric vehicles with nonmatching disturbances [38,39]. An integrated model of the suspension and steering systems was focused on designing a model predictive controller (MPC) that considered the real-time impact of uncertainties and promptly corrected them. This approach enhanced vehicle ride comfort, maneuverability, and handling stability [40,41,42]. A synchronization method based on slip ratio was introduced for the stability and driving performance of distributed drive electric vehicles in split friction regions and actuator failures under the integrated control architecture of high, medium, and low-level controllers. This method redistributes the driving forces of all drive wheels to ensure the stability and driving performance of electric vehicles in split friction regions [43]. These methods of fault-tolerant control often rely on reducing the vehicle’s velocity or altering the front wheel steering angle to maintain stability. This reliance on reducing vehicle velocity or altering the front wheel steering angle results in a reduced sense of control for the driver. At the same time, existing layered control architectures mainly focus on fault tolerance for single in-wheel motor failures. They often fail to address situations where multiple motors experience performance degradation simultaneously effectively. Additionally, most methods rely on a single system, such as the driving system, to compensate for faults, which may not be sufficient under severe failure conditions.

Therefore, this paper proposes a fault-tolerant control strategy designed for scenarios where the output capacity of in-wheel motors remains either sufficient or insufficient following a fault. First, a two-degree-of-freedom vehicle model and an in-wheel motor model are established to calculate the desired center of mass sideslip angle and yaw rate. Then, a longitudinal PID controller is constructed based on the deviation between the desired and actual vehicle speeds to calculate the required total longitudinal force. At the same time, a lateral motion MPC controller is developed to calculate the required additional yaw moment. Finally, two different driving force distribution schemes are designed based on the output capacity after in-wheel motor failure to optimize the torque distribution for the four wheels in the torque distribution layer. When the output capacity is sufficient after in-wheel motor failure, the torque distribution optimization considers only the motor’s capacity and constraints, such as road adhesion conditions. When the output capacity is insufficient after in-wheel motor failure, a multi-system collaboration distribution strategy is proposed, which coordinates the hydraulic braking system and front-wheel steering system. This strategy improves tire stability margin, ensures vehicle stability, and optimizes both driving performance and handling.

## 2. Dynamic Modeling

### 2.1. Dynamics Model of 4WDEV

**Figure 1:**
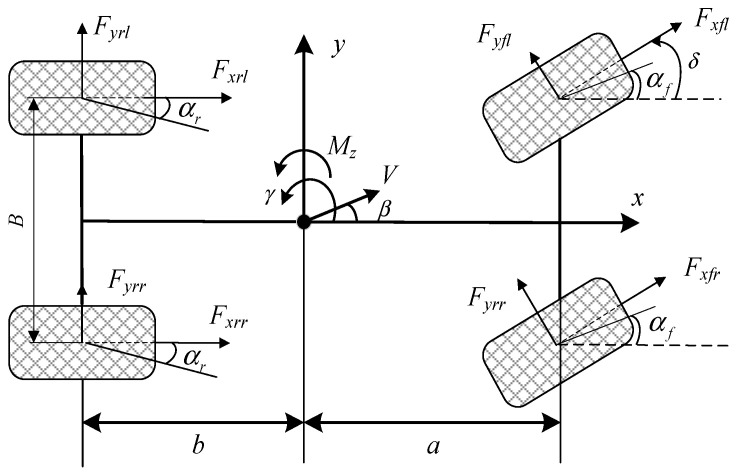
Dynamics model of 4WDEV.

The actual 4WDEV, as shown in Figure 1, is a system with strong nonlinear and high degrees of freedom, so it needs to be simplified accordingly for dynamic modeling. In this paper, four hypotheses are designed as the research framework: first, the 4WDEV body is considered as a rigid body; second, the vehicle runs on a level road surface; third, the suspension and tires are always perpendicular to the ground; finally, the sideward characteristics of the tire are always in the normal range when the vehicle is running.

The dynamic equations of the simplified 4WDEV in longitudinal, lateral, and yaw directions can be shown as [44,45]

(1) $$m({\dot{V}}_{x}-V_{y}\cdot \gamma )=(F_{xfl}+F_{xfr})\cos\delta -(F_{yfl}+F_{yfr})\sin\delta +F_{xrl}+F_{xrr}$$

(2) $$m({\dot{V}}_{y}+V_{x}\cdot \gamma )=(F_{xfl}+F_{xfr})\sin\delta +(F_{yfl}+F_{yfr})\cos\delta +F_{yrl}+F_{yrr}$$

(3) $$\begin{array}{l} I_{z}\dot{\gamma }=a\cdot [(F_{xfl}+F_{xfr})\sin\delta +(F_{yfl}+F_{yfr})\cos\delta ]-b\cdot (F_{yfl}+F_{yfr})\\ \;\;\;\;+\frac{B}{2}\cdot [(F_{xfr}-F_{xfl})\cos\delta +(F_{yfl}-F_{yfr})\sin\delta +F_{xrr}-F_{xrl}] \end{array}$$

where *V_x_* and *V_y_* represent the vehicle’s longitudinal (*x*-axis) and lateral (*y*-axis) velocities, *γ* and *δ* are the yaw rate and front-wheel steering angle of the vehicle; *F_xfl_*, *F_xfr_*, *F_yfl_,* and *F_yfr_* are the longitudinal and lateral forces from the left and right front wheels, respectively, *F_xrl_*, *F_xrr_*, *F_yrl_*, and *F_yrr_* are the longitudinal and lateral forces from the left and right rear wheels, respectively. *m* is the mass of the vehicle, *I_z_* is the moment of inertia of the vehicle about a vertical axis (*z-*axis); *a* and *b* are the distances from the vehicle’s center of mass to the front and rear axles, respectively; *B* is the wheel track.

The simplification of the tire dynamics is mainly applicable to medium-to-high adhesion road surfaces, small slip angle ranges, and steady-state driving conditions.

From the perspective of 4WDEV’s longitudinal characteristics, the longitudinal force *F_xij_* (*i* = *f*, *r*; *j = l*, *r*) of the *i*th axle and the *j*th side wheel can be linearly approximated in the Magic equation, as shown:

(4) $$F_{xij}=C_{xij}\cdot \kappa_{xij}$$

where *κ_xij_* is the longitudinal slip rate of the *i*th axle, the *j*th side wheel is the function of the angular velocity and the effective radius of the corresponding wheel, *C_xij_* is the corresponding longitudinal stiffness that is connected with the corresponding vertical load *F_zij_* and fitting coefficient.

Similarly, the lateral forces *F_yij_* (*i* = *f*, *r*; *j = l*, *r*) of the *i*th axle and the *j*th side wheel can be linearly expressed as

(5) $$F_{yij}=C_{yi}\cdot \alpha_{i}$$

where *C_yi_* (*i* = *f*, *r*) is the lateral stiffness of the *i*th axle wheel, and *α_i_* (*i* = *f*, *r*) represents the sideslip angle of the *i*th axle wheel. In general, the sideslip angles from the same axle wheels are equal, but the sideslip angles from the front and rear axles are different. Then, the sideslip angle *α_i_* of the *i*th axle wheel can be expressed as

(6) $$\alpha_{i}=\beta +\frac{a\left|\mathrm{sgn}(i-r)\right|\cdot \gamma +b\left|\mathrm{sgn}(i-f)\right|\cdot \gamma }{V_{x}}-\left|\mathrm{sgn}(i-r)\right|\cdot \delta$$

where *β* is the sideslip angle of the vehicle.

The rotation equation of the *i*th axle and the *j*th side wheel is as follows:

(7) $$J{\dot{\omega }}_{ij}=T_{dij}-T_{bij}-F_{xij}R$$

where *R* and *J* are the radius and moment of inertia of each wheel, *ω_ij_* is the angular velocity of the *i*th axle and the *j*th side wheel, and *T_dij_* and *T_bij_* are the driving torque and braking torque of the corresponding wheel.

### 2.2. In-Wheel Motor Model

In-wheel motors serve as the power input sources for 4WDEV, and the actual torque generated by each in-wheel motor is taken as the driving torque for the corresponding wheel. Given that the primary emphasis of this study lies in the vehicle stability control method, the electromagnetic conversion process of each in-wheel motor is simplified into a mathematical model, which is expressed as a second-order transfer function; the parameter selection of the model is based on the method in [46]

(8) $$G(s)=\frac{1}{2\xi^{2}s^{2}+2\xi s+1}$$

where *ζ* represents the characteristic parameter of the in-wheel motor, its value is determined by fitting corresponding experimental data. In this paper, *ζ* is set as 0.05 in the simulation of the in-wheel motor used in 4WDEV.

In general, the actual torque and expected torque of each in-wheel motor are approximately equal. When an in-wheel motor fails, the actual torque would be lower than the expected torque, and the gap widens with an increase in the fault degree. Moreover, the operating condition of each in-wheel motor is usually divided into three states: normal state, partial failure, and complete failure [47,48]. Certainly, partial failure can be divided into minor faults and severe faults. Here, the health factor *ε*_ij_ represents the operating condition of the *i*th axle and the *j*th side in-wheel motor. It is calculated based on the ratio of the actual torque to the expected torque.

(9) $$\varepsilon_{ij}=\frac{T_{mij}}{T_{mij}^{\prime }}$$

where *T_m__ij_* and *T′_mij_* (*i* = *f*, *r*; *j = l*, *r*) represent the actual torque and expected torque of the *i*th axle and the *j*th side in-wheel motor, respectively.

Obviously, the larger the value of the health factor, the smaller the fault degree of the in-wheel motor, and vice versa. Considering the possible hysteresis of the driving system and the volatility of actual monitoring data, the threshold of *ε_ij_* is set for each state of the in-wheel motor. When 0.95 ≤ *ε_ij_* ≤ 1, the in-wheel motor can be regarded as a normal state. When 0.05 ≤ *ε_ij_* < 0.95, there is speculation that partial failure has occurred. As far as what extent is a severe fault, there is no agreed standard against which to judge the fault state of the in-wheel motor. In this paper, when the health factor *ε_ij_* of any in-wheel motor is in the 0.5–0.95 range, the driving system of 4WDEV can be controlled for vehicle stability, then the output capacity of the in-wheel motor can be regarded as sufficient, the corresponding fault state can be judged as a minor fault. Otherwise, when 0.05 ≤ *ε_ij_* < 0.5, the local output capacity is insufficient, and it is difficult to control the vehicle stability, then the state of the in-wheel motor is recognized as a severe fault. When 0 ≤ *ε_ij_* < 0.05, the in-wheel motor is identified as a complete failure.

## 3. Fault-Tolerant Controller Design

**Figure 2:**
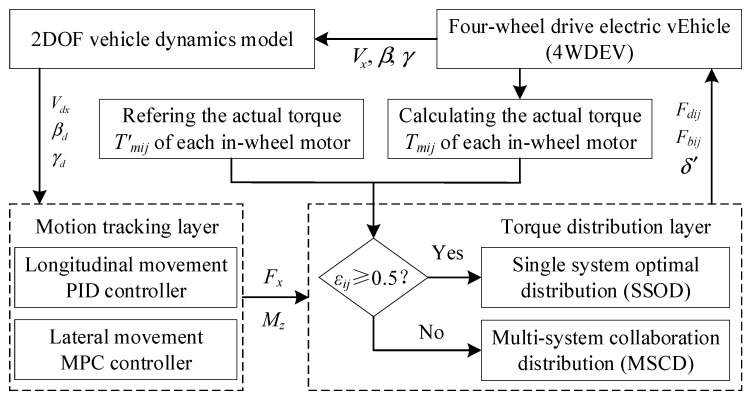
Fault-tolerant control policies of 4WDEV with one or more in-wheel motors’ faults.

In order to guarantee the safe and stable driving of 4WDEV even in the event of one or more in-wheel motor malfunctions, the control system of the 4WDEV is structured in a two-layer architecture. These two layers are the motion tracking layer and the torque distribution layer, which work in concert to maintain vehicle performance under such adverse conditions. In the motion tracking layer, a two-degree-of-freedom vehicle model is employed as a reference model for obtaining the expected yaw rate and sideslip angle of the vehicle. Then, the MPC controller is designed based on the sideslip angle and yaw rate deviation [49,50,51] to obtain the additional yaw moment *M_z_*. According to the deviation of the vehicle’s driving velocity, the PID controller is used to obtain the total longitudinal force *F_x_* required by the vehicle. The parameter settings of the MPC controller and the PID controller were referenced from [52], and appropriate adjustments were made based on it to optimize controller performance and adapt to the specific application scenario of this study. In the torque distribution layer, two control strategies are designed: single-system optimal distribution (SSOD) and multi-system collaboration distribution (MSCD), which are used to cope with the sufficient and insufficient output capabilities of one or more in-wheel motors. Fault-tolerant control policies of 4WDEV with one or more in-wheel motors’ faults are shown in Figure 2.

### 3.1. Reference Model

The two-degree-of-freedom vehicle model is a classical reference model for researching the lateral motion and yaw motion of the vehicle [53]. Certainly, it is assumed that the longitudinal velocity of the lane-following vehicle is constant, and the steering angles of the left and right wheels are equal. The vehicle model can be simplified, and the state-space equations can be derived as follows:

(10) $$\dot{x}=Ax+Bu$$

(11) y=Cx

where $x=y={\left[\beta \gamma \right]}^{T}$, $u=M_{z}$, $C=\left[\begin{array}{cc} 1 & 0\\ 0 & 1 \end{array}\right]$: $A=\left[\begin{array}{cc} -\frac{C_{yf}+C_{yr}}{mV_{x}} & \frac{C_{yr}b-C_{yf}a}{mV_{x}^{2}}-1\\ \frac{C_{yr}b-C_{yf}a}{I_{z}} & -\frac{C_{yf}a^{2}+C_{yr}b^{2}}{I_{z}V_{x}} \end{array}\right]$, $B=\left[\begin{array}{c} 0\\ \frac{1}{I_{z}} \end{array}\right]$.

Moreover, the yaw rate of the vehicle under steady-state operating conditions can be calculated by:

(12) $$\gamma_{d}^{*}=\frac{V_{x}}{\left(1+KV_{x}^{2})(a+b\right)}\delta$$

where $K=\frac{m}{L^{2}}(\frac{a}{C_{yr}}-\frac{b}{C_{yf}})$ is the stability index of the vehicle.

Taking into account the constraint of the road adhesion coefficient, as documented in [54], the vehicle’s yaw rate has an upper limit value. Consequently, the desired yaw rate can be formulated as:

(13) $$\gamma_{d}=\min(\gamma_{d}^{*},\;|\frac{\mu g}{V_{x}}|)\cdot \mathrm{sgn}(\delta )$$

where *μ* Is the road adhesion coefficient, and *g* is the acceleration of gravity.

When the sideslip angle is excessive, the possibility of vehicle instability can increase. Therefore, the expected sideslip angle *β_d_* is usually set to 0.

### 3.2. Motion Tracking Layer

#### 3.2.1. Lateral Motion Controller

The control goal of the lateral motion controller is to ensure that the vehicle’s sideslip angle fluctuates within a small range and to minimize the deviation between the actual and desired yaw rates. Compared with LQR, MPC can predict the future states of the system during the optimization process and explicitly consider input and state constraints. Additionally, MPC can be directly applied to a wider range of nonlinear control problems. Therefore, the lateral motion controller is designed by the MPC algorithm [55,56,57].

First, the forward Euler method is used to discretize the state-space equations (10) and (11) at the sampling point *k*:

(14) $$x(k+1)=\bar{A}x(k)+\bar{B}u(k)$$

(15) $$y(k)=\bar{C}x(k)$$

where $\bar{A}=I+T_{s}A$, $\bar{B}=T_{s}B$, $\bar{C}=C$, Ts is the sampling time.

Let:

(16) Δx(k)=x(k)−x(k−1)

(17) Δu(k)=u(k)−u(k−1)

Thus, Equations (14) and (15) are changed into the incremental form as shown:

(18) $$\Delta x(k+1)=\bar{A}\Delta x(k)+\bar{B}\Delta u(k)$$

(19) $$y(k)=\bar{C}\Delta x(k)+y(k-1)$$

When $\tilde{x}(k)={\left[\Delta x(k)y(k)\right]}^{T}$, a new state-space equation can be derived.

(20) $$\tilde{x}(k+1)=\tilde{A}\tilde{x}(k)+\tilde{B}\tilde{u}(k)$$

(21) $$\tilde{y}(k)=\tilde{C}\;\tilde{x}(k)$$

where $\tilde{A}=\left[\begin{array}{cc} \bar{A} & 0\\ \bar{C}\;\bar{A} & I \end{array}\right]$, $\tilde{B}=\left[\begin{array}{c} \bar{B}\\ \bar{C}\;\bar{B} \end{array}\right]$, $\tilde{C}=\left[\begin{array}{cc} 0 & I \end{array}\right]$.

Based on the basic principles of MPC and Equations (20) and (21), the state changes in the system in the *N_p_* sampling time domain can be predicted in the future, and the prediction output can be expressed as

(22) $$Y=\Psi \tilde{x}(k)+\Theta U(k)$$

where $Y=\left[\begin{array}{c} \tilde{y}(k+1|k)\\ \tilde{y}(k+2|k)\\ \vdots \\ \tilde{y}(k+N_{p}|k) \end{array}\right]$, $U=\left[\begin{array}{c} \Delta \tilde{u}(k)\\ \Delta \tilde{u}(k+1)\\ \vdots \\ \Delta \tilde{u}(k+N_{p}) \end{array}\right]$, $\Psi =\left[\begin{array}{c} \tilde{C}\tilde{A}\\ \tilde{C}{\tilde{A}}^{2}\\ \vdots \\ \tilde{C}{\tilde{A}}^{N_{p}} \end{array}\right]$, $\Theta =\left[\begin{array}{cccc} \tilde{B} & \cdots & 0 & 0\\ \tilde{A}\tilde{B} & \tilde{B} & \cdots & 0\\ \vdots & \vdots & \ddots & \vdots \\ {\tilde{A}}^{N_{p}-1}\tilde{B} & {\tilde{A}}^{N_{p}-2}\tilde{B} & \cdots & \tilde{B} \end{array}\right]$.

To ensure that the vehicle follows the reference model while maintaining safe and stable driving, the control requirements of the yaw rate and sideslip angle of the vehicle are considered comprehensively to construct the objective function as follows:

(23) $$\begin{array}{l} J_{MPC}={\left\|Y-R_{MPC}\right\|}_{Q_{MPC}}^{2}+{\left\|U\right\|}^{2}\\ \;\;=\sum_{i=1}^{N_{p}}{\left[\tilde{y}(k+i|k)-r(k+j)\right]}^{T}Q_{MPC}\left[\tilde{y}(k+i|k)-r(k+j)\right]+\sum_{i=0}^{N_{c}-1}\Delta \tilde{u}{\left(k+i|k\right)}^{T}\Delta \tilde{u}(k+i|k) \end{array}$$

where $R_{MPC}={\left[r(k)\;r(k+1)\;\cdots \;r(k+N_{c}-1)\right]}^{T}$ is the expected value of the sideslip angle and yaw rate output by the reference vehicle, $Q_{MPC}=diag(q_{\beta }q_{\gamma })$ is the diagonal matrix that consists of the weights *q_β_* and *q_γ_* of sideslip angle and yaw rate. *N_c_* is the control step. r is the expected sequence that consists of the sideslip angle and yaw rate.

The objective function of *J_MPC_* mainly consists of two parts: the first part is to ensure that the vehicle tracks the desired target as much as possible, and the second part is to control the movement as little as possible to ensure the smooth operation of the vehicle. In practical applications, some constraints must be subjected as follows:

(24) $$\left\{\begin{array}{l} u_{\min}\leq u(k+j|k)\leq u_{\max}\\ \Delta u_{\min}\leq \Delta u(k+j|k)\leq \Delta u_{\max} \end{array}\right.$$

where *j* = 1, 2, …, *N_c_* − 1. *u*_min_ and *u*_max_ are the lower limit and upper limit of the control input, respectively, which represent the maximum additional yaw moment and the minimum additional yaw moment under the current state; Δ*u*_min_ and Δ*u*_max_ are the upper limit and lower limit of the change rate of additional yaw moment.

The solution to the lateral motion controller is essentially a constrained optimization problem, so the quadratic programming algorithm is used for optimization and solution. The objective function is transformed into a quadratic programming form, and then MATLAB R2023a is used to solve the quadratic programming [58], ultimately obtaining the optimal control quantity.

#### 3.2.2. Longitudinal Motion Controller

During the driving process of 4WDEV, it is necessary to ensure that the driving velocity can change with the expected vehicle velocity. To reduce the complexity of the vehicle control system and improve stability, a PID controller is used to calculate the total longitudinal force *F_x_* [59].

(25) $$F_{x}=K_{P}\cdot e(t)+K_{I}\cdot \int e(t)+K_{D}\frac{de(t)}{dt}$$

where *e*(*t*) is the difference between the actual vehicle velocity and the expected vehicle velocity; that is, $e(t)=V_{x}-V_{dx}$; *K_P_*, *K_I,_* and *K_D_* are the proportional coefficient, integral coefficient, and differential coefficient in the PID controller, respectively.

### 3.3. Torque Distribution Layer

The torque distribution layer calculates the total longitudinal force obtained from the motion tracking layer along with additional lateral moments while considering optimization objectives and various constraint limitations. The resulting control inputs are allocated to four in-wheel motors in the different axles and sides of 4WDEV to ensure the vehicle’s stable operation even in the event of one or more in-wheel motor failures. In consideration of the output capabilities of individual in-wheel motors, two distinct distribution strategies have been developed: the Single System Optimal Distribution (SSOD) and the Multi-System Collaboration Distribution (MSCD). These strategies are tailored to address scenarios where one or more in-wheel motors exhibit either sufficient or insufficient output capabilities.

#### 3.3.1. Single System Optimal Distribution

The control objective of 4WDEV is to ensure lateral stability and dynamic performance, but some constraints, such as each in-wheel motor capacity and road surface adhesion condition, must be considered. Otherwise, the solutions of each in-wheel motor driving force deviate from the reality of in-wheel motor, and thus, it is destined to be not ideal [60,61]. When one or more in-wheel motors have minor faults, the output capabilities are sufficient, and the desired torque of each wheel can be redistributed in the driving system for the vehicle’s yaw stability and power performance. Since the driving system of 4WDEV is only used to realize the optimal solution, the method is called a ‘single system optimal distribution (SSOD)’. The objective function $J_{s}^{low}$ of SSOD in the lower layer is shown as:

(26) $$J_{s}^{low}={\left\|w_{s}^{low}(B_{s}^{low}u_{s}^{low}-y_{s}^{low})\right\|}_{2}$$

(27) $$\left\{\begin{array}{c} \varepsilon_{ij}\cdot T_{\min}\leq F_{dxij}\cdot R\leq \varepsilon_{ij}\cdot T_{\max}\\ -\sqrt{{\left(\mu F_{zij}\right)}^{2}-F_{yij}^{2}}\leq F_{dxij}\leq \sqrt{{\left(\mu F_{zi}\right)}^{2}-F_{yij}^{2}} \end{array}\right.$$

where ${\left\|\cdot \right\|}_{2}$ is the 2-norm of a matrix, $u_{s}^{low}$ is the real state matrix of control variables that consist of the driving force from four wheels $u_{s}^{low}={\left[F_{dfl}\;F_{dfr}\;F_{drl}\;F_{drr}\right]}^{T}$. $w_{s}^{low}$ is the weight coefficient matrix of longitudinal force *F_x_* and yaw moment *M_z_*. *μ* is the road adhesion coefficient, and *F_zij_* is the vertical load of each wheel. $y_{s}^{low}$ is the expected state matrix of the whole vehicle and $B_{s}^{low}$ is the coefficient matrix of the driving forces from four wheels, as follows:

(28) $$y_{s}^{low}={\left[\begin{array}{cc} F_{x} & M_{z} \end{array}\right]}^{T}$$

(29) $$B_{s}^{low}=\left[\begin{array}{cccc} \cos\delta & \cos\delta & 1 & 1\\ \frac{B}{2}\cos\delta & -\frac{B}{2}\cos\delta & \frac{B}{2} & -\frac{B}{2} \end{array}\right]$$

#### 3.3.2. Multi-System Collaboration Distribution

When one or more in-wheel motors have severe faults, the allocation of driving forces in the whole vehicle must result in an increased torque requirement for the motors on the same side as the malfunctioning motor, and the required driving force becomes excessive in such conditions. It may lead to a situation that which the output capabilities of the corresponding motors are insufficient; a serious situation might cause the motors to burn out or reduce torque output in a short period [62,63]. In such a scenario, maintaining the vehicle’s dynamic performance unchanged through torque distribution based solely on in-wheel motor driving forces is not sufficient to keep the vehicle in a stable state. Therefore, a new idea is proposed that three systems of driving, braking, and front-wheel steering are combined to compensate for local missing torque and the required yaw moment, which approach is called ‘multi-system collaboration distribution (MSCD)’. With the involvement of the braking and front-wheel steering systems, the vehicle dynamic of 4WDEV must change; the dynamic equation can be expressed as follows:

(30) $$\left\{\begin{array}{l} \begin{array}{l} F_{x}=(F_{dfl}-F_{bfl})\cos\delta_{f}+(F_{dfr}-F_{bfr})\cos\delta_{f}\\ \;\;+F_{drl}-F_{brl}+F_{drr}-F_{brr}-(F_{yfl}+F_{yfr})\sin\delta_{f} \end{array}\\ \begin{array}{l} M_{z}=\frac{B_{f}}{2}[(F_{dfl}-F_{bfl})\cos\delta_{f}+(F_{dfr}-F_{bfr})\cos\delta_{f}+F_{drl}\\ \;\;-F_{brl}+F_{drr}-F_{brr}+(F_{yfl}-F_{yfr})\sin\delta_{f}]-aC_{f}\Delta \delta_{f} \end{array} \end{array}\right.$$

where $\Delta \delta_{f}$ is the additional steering angle applied to the front wheel steering system, *F_bij_* is the braking force of each wheel, and *F_dij_* is the driving force of each wheel. *F_yfl_* and *F_yfr_* are the lateral forces of the left and right front wheels, respectively, where the steering angle is $\delta_{f}^{\prime }=\delta_{f}+\Delta \delta_{f}$.

Since the focus is primarily on the distribution of the additional yaw moment, the effect of the lateral force is relatively small and thus neglected, resulting in the following equation.

(31) $$\left\{\begin{array}{l} \begin{array}{l} F_{x}=(F_{dfl}-F_{bfl})\cos\delta_{f}+(F_{dfr}-F_{bfr})\cos\delta_{f}\\ \;\;+F_{drl}-F_{brl}+F_{drr}-F_{brr} \end{array}\\ \begin{array}{l} M_{z}=\frac{B_{f}}{2}[(F_{dfl}-F_{bfl})\cos\delta_{f}+(F_{dfr}-F_{bfr})\cos\delta_{f}\\ \;\;\;+F_{drl}-F_{brl}+F_{drr}-F_{brr}]-aC_{f}\Delta \delta_{f} \end{array} \end{array}\right.$$

Taking into account the possible output capability of each in-wheel motor, the fluctuation range of the additional front-wheel angle, and the road adhesion conditions, the objective functions $J_{m}^{low}$ of MSCD in the lower layer are listed:

(32) $$J_{m}^{low}={\left\|w_{m}^{low}(B_{m}^{low}u_{m}^{low}-y_{m}^{low})\right\|}_{2}$$

(33) $$s.t.\;\left\{\begin{array}{c} \begin{array}{l} \varepsilon_{ij}\cdot T_{\min}\leq F_{dxij}\cdot R\leq \varepsilon_{ij}\cdot T_{\max}\\ \Delta \delta_{\min}\leq \Delta \delta \leq \Delta \delta_{\max} \end{array}\\ -\sqrt{{\left(\mu F_{zij}\right)}^{2}-F_{yij}^{2}}\leq F_{dxij}\leq \sqrt{{\left(\mu F_{zij}\right)}^{2}-F_{yij}^{2}} \end{array}\right.$$

where $w_{m}^{low}$ is the weight coefficient matrix of the control quantity. $u_{m}^{low}$ is the real state matrix of control variables that consist of the driving force, braking force from four wheels, and the additional steering angle of the front wheels $u_{m}^{low}={\left[F_{dfl}\;F_{dfr}\;F_{drl}\;F_{drr}\;F_{dfl}\;F_{dfr}\;F_{drl}\;F_{drr}\;\Delta \delta \right]}^{T}$. $y_{m}^{low}$ is the expected state matrix of the whole vehicle, and $B_{m}^{low}$ is the coefficient matrix of the driving force, braking force from four wheels, and the additional steering angle of the front wheels, as follows:

(34) $$y_{m}^{low}={\left[\begin{array}{cc} {F^{\prime }}_{x} & {M^{\prime }}_{z} \end{array}\right]}^{T}$$

(35) $$B_{m}^{low}=\left[\begin{array}{ccc} E & F & G \end{array}\right]$$

where $E=\rho_{1}\left[\begin{array}{llll} \cos\delta & \cos\delta & 1 & 1\\ -\frac{B}{2}\cos\delta & \frac{B}{2}\cos\delta & -\frac{B}{2} & \frac{B}{2} \end{array}\right]$, $F=\rho_{2}\left[\begin{array}{llll} -\cos\delta & -\cos\delta & -1 & -1\\ \frac{B}{2}\cos\delta & -\frac{B}{2}\cos\delta & \frac{B}{2} & -\frac{B}{2} \end{array}\right]$, $G=\rho_{3}\left[\begin{array}{c} 0\\ -a\;C_{yf} \end{array}\right]$. *ρ*1, *ρ*2, and *ρ*3 are the control distribution coefficients of the driving system, braking system, and front-wheel steering system, and the sum of the three values is 1.

By organizing the objective functions (26) and (32) into a quadratic programming form and solving the quadratic programming using MATLAB, the optimal control quantity of each in-wheel motor in the lower layer can be obtained.

### 3.4. Vehicle State Observer Design

The unscented Kalman filter (UKF) is an improved filtering method developed from the extended Kalman filter (EKF), designed to address the issue where the observation performance of EKF degrades as nonlinearity increases. The UKF improves filtering accuracy and stability by generating a set of sigma points to sample the system, which better describes and captures the nonlinearity of the model.

Based on the dynamics modeling of the 4WDEV and the in-wheel motor model, the estimation model of the in-wheel motor health factor is represented by the following equation.

(36) $$\varepsilon_{ij}=\frac{1}{T_{mij}^{\prime }}[I_{w}{\dot{\omega }}_{ij}+RF_{xij}+F_{zij}R]$$

In the design of the 4WDEV state observer, the longitudinal velocity, lateral velocity, yaw rate, and rotational speed of the electric wheels are selected as the state variables of the system, specifically as follows:

(37) $$x(t)={\left[V_{x},V_{y},\gamma ,\omega_{fl},\omega_{fr},\omega_{rl},\omega_{rr}\right]}^{T}$$

The control inputs are chosen as the output torques of the four in-wheel motors, namely:

(38) $$u(t)={\left[T_{dfl},T_{dfr},T_{drl},T_{drr}\right]}^{T}$$

It is assumed that the vehicle’s longitudinal and lateral accelerations can be measured by the onboard inertial measurement unit. Based on these assumptions, the following parameters are taken as the outputs of the model:

(39) $$y(t)={\left[a_{x},a_{y},\gamma ,\omega_{fl},\omega_{fr},\omega_{rl},\omega_{rr}\right]}^{T}$$

The vehicle’s state-space equations are established as follows:

(40) $$\left\{\begin{array}{l} \dot{x}=f(x)+Bu\\ y=g(x) \end{array}\right.$$

where the front wheel steering angle δ is considered as a disturbance input to the system, and this parameter changes over time.

(41) $$B=\frac{1}{I_{w}}{\left[\begin{array}{ccccccc} 0 & 0 & 0 & 0 & 0 & 0 & \varepsilon_{rr}\\ 0 & 0 & 0 & 0 & 0 & \varepsilon_{rl} & 0\\ 0 & 0 & 0 & 0 & \varepsilon_{fr} & 0 & 0\\ 0 & 0 & 0 & \varepsilon_{fl} & 0 & 0 & 0 \end{array}\right]}^{T}$$

The system state variable matrix is expanded as follows:

(42) $$\tilde{x}={\left[V_{x},V_{y},\gamma ,\omega_{fl},\omega_{fr},\omega_{rl},\omega_{rr},\varepsilon_{fl},\varepsilon_{fr},\varepsilon_{rl},\varepsilon_{rr}\right]}^{T}$$

The system is discretized using the forward Euler method, and the discretized state-space equations are as follows:

(43) $$\left\{\begin{array}{l} \tilde{x}(k+1)=\tilde{f}(\tilde{x}(k))+T_{s}\tilde{B}u(k)+v(k)\\ y=\tilde{g}(\tilde{x}(k))+w(k) \end{array}\right.$$

where *v*(*k*) is the system noise, *w*(*k*) is the measurement noise, and *T_s_* is the sampling time for the Euler discretization. The system input matrix $\tilde{B}$ is:

(44) $$\tilde{B}=\frac{1}{I_{w}}{\left[\begin{array}{ccccccccccc} 0 & 0 & 0 & 0 & 0 & 0 & \varepsilon_{rr} & 0 & 0 & 0 & 0\\ 0 & 0 & 0 & 0 & 0 & \varepsilon_{rl} & 0 & 0 & 0 & 0 & 0\\ 0 & 0 & 0 & 0 & \varepsilon_{fr} & 0 & 0 & 0 & 0 & 0 & 0\\ 0 & 0 & 0 & \varepsilon_{fl} & 0 & 0 & 0 & 0 & 0 & 0 & 0 \end{array}\right]}^{T}$$

By incorporating the in-wheel motor health factor into part of the system state through the above equations, real-time estimation of the in-wheel motor health factor is achieved, enabling the online identification of the in-wheel motor health factor parameters.

## 4. Performance Verification

To validate the effectiveness of the fault-tolerant control strategy proposed in this paper for 4WDEV with one or more in-wheel motors’ faults, a combined simulation model was developed using Simulink/MATLAB and CarSim 2022.1. CarSim is suitable for control algorithm validation that requires high accuracy while focusing on computational efficiency, achieving a good balance between dynamic simulation and computation time. Additionally, CarSim provides a rich set of vehicle dynamics models, supports direct modification of parameters, such as mass, tires, suspension, etc., and offers a visual interface, making it easy to adjust and validate control strategies. Therefore, CarSim simulation software was chosen. The simulation experiment was conducted on a computer with an Intel(R) Core(TM) i7-8750H CPU @ 2.20GHz, 8.00 GB of RAM, and a 64-bit Windows 10 operating system. During runtime, other nonessential programs were closed to ensure that system resources were primarily allocated to the simulation experiment.

The overall vehicle dynamics model adopts the C-class car model from CarSim, and the control algorithms were implemented using Simulink. Moreover, simulation analysis was conducted to investigate the vehicle’s operating states under two scenarios: the firster is that the output capacity of each in-wheel motor is sufficient, and the seconder is that the output capacities of one or more in-wheel motors are insufficient [64]. The primary parameters of 4WDEV are listed in Table 1, and the primary parameters of the simulation are listed in Table 2.

**Figure 3:**
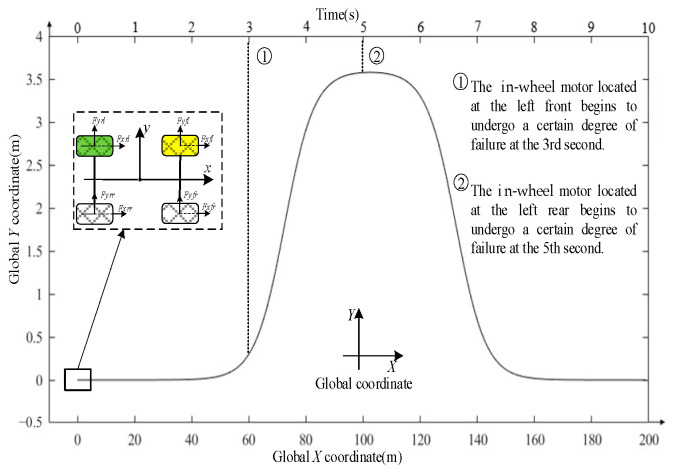
Schematic of the simulation environment.

The simulation environment includes a straight-line track environment and a DLC track environment. Taking the DLC track environment as an example, the global coordinate map of the vehicle’s trajectory is shown in Figure 3 (*X*–*Y* Global coordinate). In the figure, the simulated vehicle starts from point 0 and moves along the *X*-axis direction, with the positions and times of in-wheel motor malfunctions explicitly marked. These correspond precisely to the simulation experiments described later in this paper.

### 4.1. Sufficient Output Capacity of Each In-Wheel Motor

**Figure 4:**
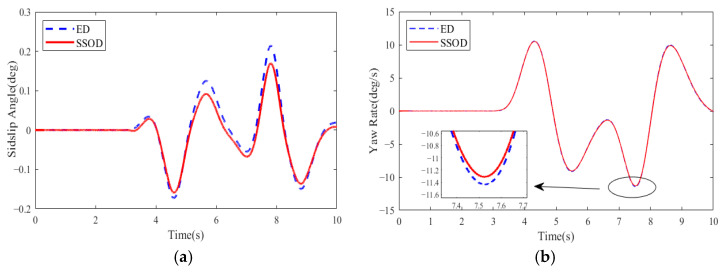
Stability indicators of two torque distribution schemes in the first test scenario: (**a**) Sideslip angle, (**b**) Yaw rate.

**Figure 5:**
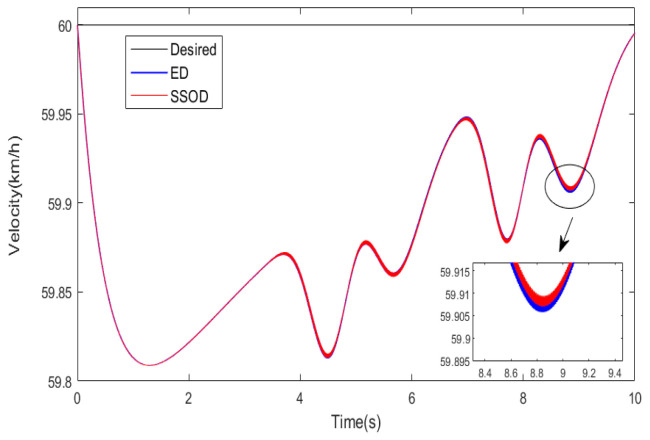
Actual vehicle velocity of 4WDEV in the first test scenario.

The general idea of the first test scenario is that a partial failure of one in-wheel motor occurs after 4WDEV has operated normally for some time. Specific information is that the desired vehicle velocity of 4WDEV was set to 60 km·h^−1^, and the road adhesion coefficient was assumed to be 0.85. One in-wheel motor located in the left front of 4WDEV started working fine, but the health factor of 60% always occurred from the 3rd second The other in-wheel motors had always been normal. In this work, the health factor of the left front in-wheel motor is 60%, and the other health factors are close to 100%. Then, the output capacity of each in-wheel motor is sufficient; the SSOD scheme can be performed under the test track of double lane change (DLC). Moreover, an equal distribution (ED) scheme that the additional yaw torque in the motion tracking layer is equally distributed for four in-wheel motors is employed to execute in the same test scenario. The results of the two torque distribution schemes are depicted as shown in Figure 4 and Figure 5.

Obviously, the sideslip angle of 4WDEV by SSOD is smaller than ED, and the maximum reduction is 25.1%. The 25.1% reduction in the sideslip angle is based on a comparison of the maximum sideslip angles, where *M* is the maximum sideslip angle with the SSOD method, and *N* is the maximum sideslip angle with the ED method. The calculation formula is as follows: $\left(N-M\right)/N*100\%$. As for the yaw rate and vehicle velocity of 4WDEV, the performances of the two torque distribution schemes are almost the same; SSOD performs slightly better than ED in some regions. By quantitative comparison with ED, SSOD has reduced the yaw rate by 5.7% and enhanced the vehicle velocity by 0.4 km·h^−1^. Certainly, two torque distribution schemes can quickly achieve the expected vehicle velocity, and the errors are controlled at 0.3%. In summary, while a failure happens on one in-wheel motor, SSOD can significantly improve vehicle stability on the basis of better vehicle dynamics if the output capacity of each in-wheel motor is sufficient.

**Figure 6:**
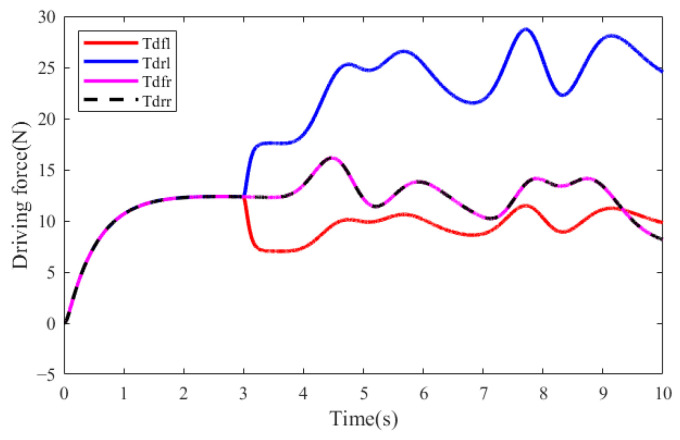
Actual driving forces of four in-wheel motors in the first test scenario.

To explore the actual distribution of driving forces by SSOD method, the force of each in-wheel motor was observed in the first test scenario, as shown in Figure 6. It is evident that the driving force of the left rear in-wheel motor swiftly increases from the 3rd second This occurs because the left front in-wheel motor malfunctions, leading to a reduction in its driving force, which in turn causes a rapid decrease in the total driving force on the left side of the vehicle. At the same time, it is judged that the health factor of the left front in-wheel motor is greater than 0.5, the left rear in-wheel motor can be utilized to compensate for the lacking force on the left side of the vehicle. Then SSOD quickly optimizes the driving force distribution of the front and rear in-wheel motors on the vehicle’s left side and remains the driving forces of two in-wheel motors on the vehicle’s right side unchanged, then ensures the total longitudinal force and the additional lateral moment required by the vehicle, and maintains the vehicle’s dynamic performance and handling stability.

### 4.2. Insufficient Output Capacities of One or More In-Wheel Motors

The second test scenario is that the severe failures of two in-wheel motors on one side of the vehicle occur in succession after 4WDEV has operated normally for some time. Specific experimental establishment is that the road adhesion coefficient was set at 0.85, four in-wheel motors of 4WDEV started working fine, but the left front in-wheel motor experienced a complete loss of functionality from the 3rd second, and the left rear in-wheel motor experienced a 60% fault from the 5th second. That is, the health factors of the left front and left rear in-wheel motors are 0 and 40%, and the health factors of the two in-wheel motors on the right side of the vehicle are close to 100%. It is obvious that the output capacities of two in-wheel motors on the left side of the vehicle are insufficient. To validate the effectiveness of the proposed MSCD method, two test tracks were designed that were a straight-line environment and a DLC environment. Moreover, the control strategy in the torque distribution layer was substituted with dual-system collaboration distribution 1 (DSCD1), cooperative allocation of steering and driving systems, and dual-system collaboration distribution 2 (DSCD2): cooperative allocation of braking and driving systems, to facilitate the comparative analysis of test outcomes. Equations (45) and (49) represent the objective functions *J*_cb_ and *J*_cs_ corresponding to the DSCD1 and DSCD2 strategies, respectively.

(45) $$J_{\mathrm{cb}}={\left\|w_{\mathrm{cb}}(B_{\mathrm{cb}}u_{\mathrm{cb}}-y_{\mathrm{cb}})\right\|}_{2}$$

(46) $$B_{\mathrm{cb}}=\left({Β}_{m1},B_{m2}\right)$$

(47) $$u_{\mathrm{cb}}={\left(F_{d},F_{b}\right)}^{T}$$

(48) $$y_{\mathrm{cb}}={\left(F_{x}\;,\;M_{z}\right)}^{T}$$

(49) $$J_{\mathrm{cs}}={\left\|w_{\mathrm{cs}}(B_{\mathrm{cs}}u_{\mathrm{cs}}-y_{\mathrm{cs}})\right\|}_{2}$$

(50) $$B_{\mathrm{cs}}=\left(B_{m1},B_{m3}\right)$$

(51) $$u_{\mathrm{cs}}={\left(F_{d}\;,\;\Delta \delta_{f}\right)}^{T}$$

(52) $$y_{\mathrm{cs}}={\left(F_{x}\;,\;M_{z}\right)}^{T}$$

where $u_{\mathrm{cb}}$ is the control input matrix for the DSCD1 strategy; $w_{\mathrm{cb}}$ is the weight coefficient matrix for the DSCD1 strategy; $y_{\mathrm{cb}}$ and $B_{\mathrm{cb}}$ are the expected state matrix and control coefficient matrix for the entire vehicle, respectively; $u_{\mathrm{cs}}$ is the control input matrix for the DSCD2 strategy; $w_{\mathrm{cs}}$ is the weight coefficient matrix for the DSCD2 strategy; $y_{\mathrm{cs}}$ and $B_{\mathrm{cs}}$ are the expected state matrix and control coefficient matrix for the entire vehicle, respectively.

#### 4.2.1. Test Analysis in a Straight-Line Track

**Figure 7:**
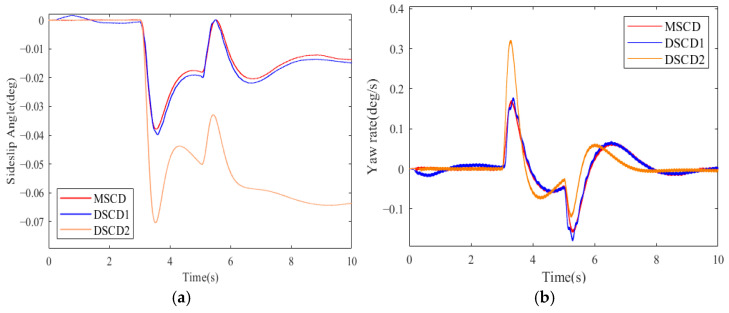
Vehicle stability indicators of three torque distribution schemes in the second test scenario and a straight-line track: (**a**) Sideslip angle (**b**) Yaw rate.

**Figure 8:**
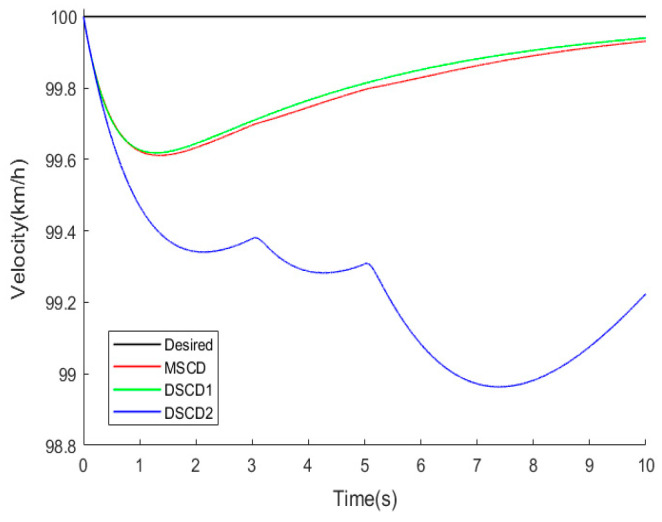
Actual vehicle velocity of three torque distribution schemes in the second test scenario and a straight-line track.

In a straight-line track, the desired vehicle velocity was set to 100 km·h^−1^. All parameters of 4WDEV remain exactly the same; three torque distribution schemes of MSCD, DSCD1, and DSCD2 were performed successively in the same test scenario. The results of the sideslip angle, yaw rate, and actual vehicle velocity are shown in Figure 7 and Figure 8.

Observe the variations in the center of mass sideslip angle and yaw rate in Figure 7. From 3.5 s to 5 s and after 6 s, it can be observed that the MSCD scheme is the optimal choice, which stems from the superiority of the three-system collaboration in the MSCD scheme. Though the stability of 4WDEV by MSCD could fluctuate at the moment of a sudden loss of output capacities, the amplitude of the fluctuation was the smallest of than two schemes of DSCD1 and DSCD2, and the duration was the shortest. Certainly, the performance of DSCD1 was close to MSCD, but DSCD2 was relatively a little worse for vehicle stability. Similarly, the actual vehicle velocities in Figure 8 were observed to find that MSCD was optimal, followed by DSCD1, then DSCD2 was proved to be poor, but MSCD and DSCD1 were not significantly different.

**Figure 9:**
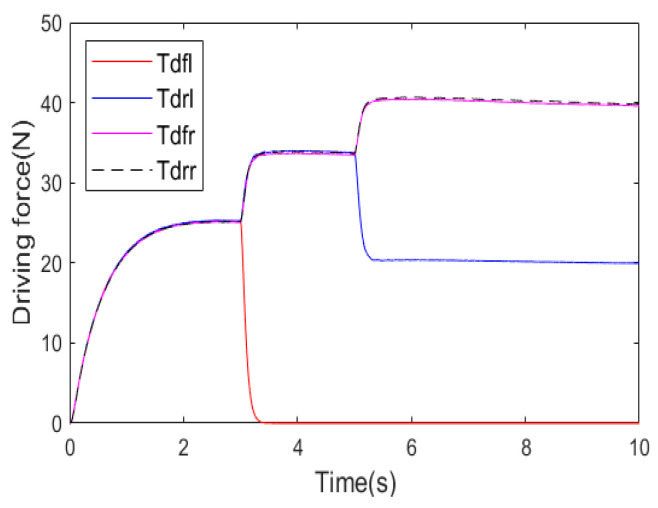
Actual driving forces of four in-wheel motors in the second test scenario and a straight-line track.

Figure 9 is the actual driving forces of four in-wheel motors in the second test scenario and a straight-line track. When the left front in-wheel motor completely became inoperative from the 3rd second, the other in-wheel motors quickly increased the driving forces to compensate for the total longitudinal force of the vehicle. At the same time, the braking and front-wheel steering systems also contributed to the driving system to ensure the required additional lateral moment of 4WDEV. According to the vehicle stability indicators, as shown in Figure 7, it is evident that 4WDEV quickly regained relative stability. At the 5th second, the left rear in-wheel motor experienced a health factor of 60%, and the output capacities on the left side of the vehicle remained significantly inadequate. Two in-wheel motors on the right side of the vehicle increased the driving forces again, and the driving system of the whole vehicle was ably assisted by the braking and front-wheel steering systems. Then, the required total longitudinal force and additional lateral moment of 4WDEV could be met. Figure 7 and Figure 8 indicate that there is some fluctuation in the sideslip angle and the yaw rate, but the stability of the whole vehicle is quickly restored, and, more importantly, the error between the actual and desired vehicle velocity stays below 0.2 km·h^−1^.

To further quantify the performance of MSCD in the second test scenario and a straight-line track, the average errors and maximum deviations of the sideslip angle and yaw rate were selected to evaluate the actual vehicle driving states, as shown in Table 3. Here, the average errors of the center of mass sideslip angle and yaw rate are obtained by averaging the errors of all data points in Figure 7 and Figure 8. Clearly, the corresponding evaluation indexes by MSCD were smaller than DSCD1 and DSCD2, especially in the average errors of the sideslip angle and yaw rate. MSCD exhibited the corresponding reductions of 6.25% and 5.77% than DSCD1, respectively. Relative to the DSCD2 scheme, MSCD lowered the average errors of the sideslip angle and yaw rate by 68.1% and 22.2%, respectively. As for the average actual vehicle velocity, there were no significant differences among the three torque distribution schemes, then they were considered as the same effect.

To enhance the reliability of the experimental conclusions, we conducted five independent simulation experiments under the same conditions. Statistical analysis was performed on key indicators, such as the average error of the vehicle’s center of mass sideslip angle, the average error of the yaw rate, and the average error of the actual vehicle velocity. The mean values and standard deviations were calculated, and relevant data are presented in Table 4. Specifically, in terms of the average errors of the sideslip angle and yaw rate, MSCD reduced them by 12.9% and 5.88%, respectively, compared with DSCD1.

The results indicate that although individual experiments may be affected by factors such as numerical computation methods, the overall trend remains consistent, thereby validating the reliability of the research conclusions.

#### 4.2.2. Test Analysis in a DLC Track

**Figure 10:**
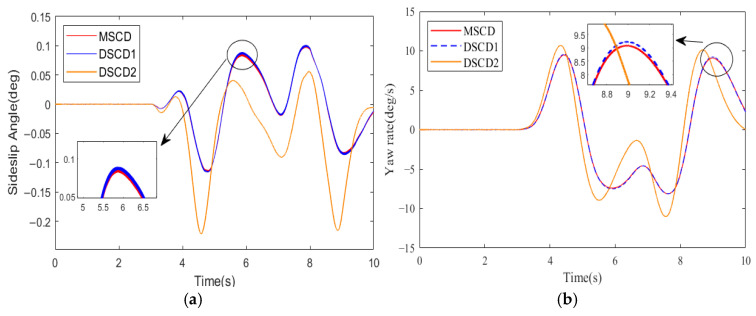
Vehicle stability indicators of three torque distribution schemes in the second test scenario and a DLC track: (**a**) Sideslip angle, (**b**) Yaw rate.

**Figure 11:**
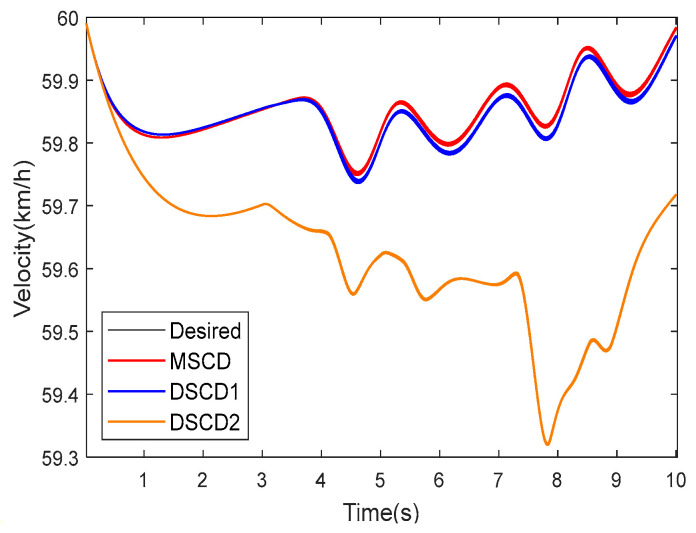
Actual vehicle velocity of three torque distribution schemes in the second test scenario and a DLC track.

In consideration of a DLC track, the desired vehicle velocity was set to 60 km·h^−1^. 4WDEV with the above parameters was used to execute three torque distribution schemes of MSCD, DSCD1, and DSCD2 in the second test scenario, respectively. The results of the sideslip angle, yaw rate, and actual vehicle velocity are shown in Figure 10 and Figure 11; the evaluation indexes of the vehicle stability and dynamic performance are summarized in Table 4.

Observations indicate that within the time range of 4.5 s to 8 s, the control performance of MSCD on the DLC trajectory is superior to that of DSCD1 and DSCD2, which stems from the superiority of the three-system collaboration in the MSCD scheme. However, MSCD took no remarkable superiority relative to DSCD1. In detail, MSCD and DSCD1 were capable of controlling the sideslip angle and vehicle velocity within a smaller range of fluctuations, while DSCD2 exhibited slightly inferior performance. Compared with the straight-line track, the fluctuations in the sideslip angle and the yaw rate of 4WDEV using any torque distribution scheme were more pronounced in the DLC tracks.

**Figure 12:**
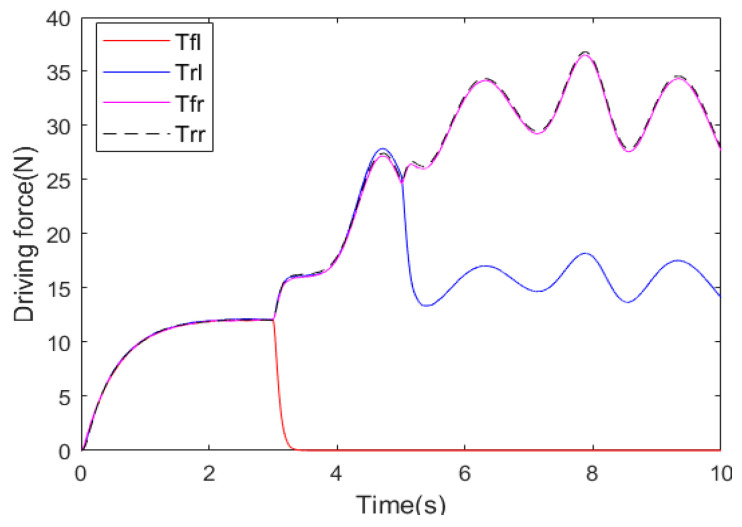
Actual driving forces of four in-wheel motors in the second test scenario and a DLC track.

Figure 12 shows the actual driving forces of four in-wheel motors in the second test scenario and the DLC track. When the left front in-wheel motor completely became inoperative from the 3rd second, the other in-wheel motors quickly increased the driving forces with the help of the braking and front-wheel steering systems, the total longitudinal force and the required additional lateral moment of 4WDEV were quickly ensured. Although 4WDEV was operating on a DLC track, the relative stability was regained. At the 5th second, the left rear in-wheel motor experienced a health factor of 60%, and the output capacities on the left side of the vehicle remained significantly inadequate. Two in-wheel motors on the right side increased the driving forces again with the help of the braking and front-wheel steering systems; the total longitudinal force and the required additional lateral moment of 4WDEV were still ensured, and the whole vehicle remained relatively stable. However, the output capacity of each in-wheel motor fluctuated more, although only the desired vehicle velocity with 60 km·h^−1^.

Similarly, the average errors and maximum deviations of the sideslip angle and yaw rate were selected to evaluate the actual vehicle driving states, as shown in Table 5. It was obvious that the five evaluation indexes by MSCD were optimal, followed by DSCD1, then DSCD2 last. MSCD had lowered the average errors of the sideslip angle and yaw rate by 7.84% and 4.59% than DSCD1, respectively, which did not show a significant advantage but could offer great reductions in the average errors of the sideslip angle and yaw rate by 35.62% and 7.71% than DSCD2.

To ensure the reliability of the experimental results, we conducted five independent simulation experiments under the same conditions and calculated the mean and standard deviation of the average error of the vehicle’s center of mass sideslip angle, the average error of the yaw rate, and the average error of the actual driving speed across the five sets of data. Relevant data are presented in Table 6. Among them, the MSCD scheme proposed in this paper achieves the best performance in the main evaluation indexes, followed by the DSCD1 scheme. Compared with the DSCD1 scheme, the MSCD scheme reduces the average errors of the center of mass sideslip angle and yaw rate by 6% and 4.5%, respectively.

Thus, it is evident that MSCD can meet the required total longitudinal force and additional lateral moment of 4WDEV to ensure the handling stability and dynamic performance of the whole vehicle in the second test scenario in both a straight-line track and a DLC track.

## 5. Conclusions

The superiority of the method proposed in the paper can be summarized by the following points:

(1) An MPC controller is used for the vehicle’s lateral motion, outputting the required additional yaw moment, while a PID controller is used for the vehicle’s longitudinal motion, outputting the required longitudinal force. This originates from the strong flexibility and adaptability of the PID controller and the predictability of the MPC controller.
(2) Novel torque distribution schemes of SSOD and MSCD are proposed on the basis of three systems of driving, braking, and front-wheel steering to deal with two scenarios of sufficient and insufficient output capacities, respectively. As a result, When the output capacity is sufficient, compared with ED, SSOD reduces the average errors of the center of mass sideslip angle and yaw rate by 25.1% and 5.7%, respectively. When the output capacity is insufficient, in the straight-line track environment, compared with DSCD1 and DSCD2, MSCD reduces the average errors of the sideslip angle and yaw rate by at least 12.9% and 5.88%, respectively. In the DLC track environment, compared with DSCD1 and DSCD2, MSCD reduces the average errors of the sideslip angle and yaw rate by at least 6% and 4.5%, respectively. The proposed SSOD and MSCD distribution strategies not only keep the vehicle’s center of mass sideslip angle and yaw rate within a small fluctuation range but also ensure good driving performance.
(3) The fault-tolerant collaborative control method with two layers of motion tracking and torque distribution is well-suited for the overall control system of 4WDEV. It lays the foundation for addressing safety concerns stemming from one or more in-wheel motors’ faults in the field of 4WDEV.

The effectiveness of the fault-tolerant collaborative control method has been preliminarily validated in this study. Although the proposed method is effective, several limitations remain. First, the vehicle model is overly simplified. Future research will incorporate the effects of dynamic load transfer, road slope, and suspension systems to enhance the model’s realism and applicability. Additionally, electromagnetic and thermal faults are crucial to the long-term operational stability of the motor. A more detailed motor health assessment model will be developed to analyze the impact of these faults on the control strategy. Lastly, this study does not consider the distribution ratio of driving forces between the front and rear axles. Future work will focus on optimizing the front-to-rear axle force distribution using advanced optimization techniques such as genetic algorithms and deep reinforcement learning. In summary, future studies will focus on more accurate vehicle modeling, a more comprehensive analysis of fault impacts, real-time performance testing of control algorithms, and optimized driving force distribution strategies to validate further the practical feasibility and engineering applicability of the proposed method.

## Figures and Tables

**Table 1:** Main parameters of 4WDEV.

Parameter	Meaning	Value
*m*	Mass of 4WDEV	1412 kg
*a*	Distance from the vehicle’s center of mass to the front axle	1.015 m
*b*	Distance from the vehicle’s center of mass to the rear axle	1.895 m
*I_z_*	Moment of inertia of the vehicle about the vertical axis (*z*-axis)	1536.7 kg·m^2^
*B*	Wheel track	1.48 m
*C_yf_*	Lateral stiffness of the front axle wheel	83,000 N·rad
*C_yr_*	Lateral stiffness of the rear axle wheel	70,000 N·rad

**Table 2:** Main parameters of the simulation.

Meaning	Value
Sampling frequency	1000 Hz
Simulation duration	10 s
Ground adhesion coefficient	0.85

**Table 3:** Evaluation indexes of the actual vehicle driving states in the second test scenario and a straight-line track.

Evaluation Index	MSCD	DSCD1	DSCD2
Average error of sideslip angle (°)	0.015	0.016	0.047
Average error of yaw rate (°·S^−1^)	0.049	0.052	0.063
Average actual vehicle velocity (km·h^−1^)	99.788	99.801	99.240
Maximum deviation of sideslip angle (°)	0.037	0.040	0.070
Maximum deviation of yaw rate (°·S^−1^)	0.166	0.178	0.318

**Table 4:** Main evaluation indexes of the actual vehicle driving states in the straight-line track.

Evaluation Index	Statistical Metrics	MSCD	DSCD1	DSCD2
Average error of sideslip angle	Mean (°)	0.0148	0.017	0.048
	Standard deviation	0.0016	0.008	0.015
Average error of yaw rate	Mean (°·S^−1^)	0.048	0.051	0.0626
	Standard deviation	0.005	0.003	0.008
Average actual vehicle velocity	Mean (km·h^−1^)	99.784	99.798	99.24
	Standard deviation	0.02	0.15	0.105

**Table 5:** Evaluation indexes of the actual vehicle driving states in the second test scenario and a DLC track.

Evaluation Index	MSCD	DSCD1	DSCD2
Average error of sideslip angle (°)	0.047	0.051	0.073
Average error of yaw rate (°·S^−1^)	5.013	5.254	5.432
Average actual vehicle velocity (km·h^−1^)	59.854	59.845	59.626
Maximum deviation of sideslip angle (°)	0.112	0.114	0.221
Maximum deviation of yaw rate (°·S^−1^)	9.062	9.209	10.130

**Table 6:** Main evaluation indexes of the actual vehicle driving states in the DLC track.

Evaluation Index	Statistical Metrics	MSCD	DSCD1	DSCD2
Average error of sideslip angle	Mean (°)	0.047	0.05	0.071
	Standard deviation	0.014	0.015	0.012
Average error of yaw rate	Mean (°·S^−1^)	5.014	5.252	5.428
	Standard deviation	0.012	0.026	0.0025
Average actual vehicle velocity	Mean (km·h^−1^)	59.852	59.836	59.614
	Standard deviation	0.005	0.055	0.043

## Data Availability

Authors declare that no data has been published.
